# Investments in health and mortality reduction to address population decline

**DOI:** 10.2471/BLT.25.293627

**Published:** 2025-12-02

**Authors:** Stuart Gietel-Basten, Wiraporn Pothisiri, Sergei Scherbov

**Affiliations:** a Divisions of Social Science and Public Policy, The Hong Kong University of Science and Technology, Hong Kong, China.; b College of Population Studies, Chulalongkorn University, Bangkok, Thailand.; c Population and Just Societies Program, International Institute for Applied Systems Analysis, Laxenburg, Austria.

## Abstract

Faced with significant population decline, many governments have turned to pronatalist policies to boost birth rates, even though such approaches are frequently ineffective and potentially infringe on reproductive rights. This study demonstrates that a more effective and immediate policy alternative exists: reducing preventable and treatable mortality. Using United Nations data, we modelled population projections to 2050 in 28 countries and territories, comparing a baseline scenario against two benchmarks: an immediate increase to replacement-level fertility and the reduction of national mortality rates to match the rate of Japan. Our findings show that investing in health is a more effective way to reduce population decline than raising fertility, particularly for countries in eastern and south-eastern Europe. For countries in the World Health Organization European Region that are most affected by population decline, achieving Japanese mortality levels would almost halve population loss, greatly outperforming the reduced decline expected with a replacement fertility approach. We consider that reducing mortality should be a central pillar of a demographic strategy. This approach offers faster demographic returns, aligns with human rights and healthy ageing goals, and provides a stronger return on prior societal investments in education and health. We recommend that policy-makers therefore move towards strengthening health systems, disease prevention and public health interventions. At the same time, they should integrate these measures with broader institutional reforms for a more sustainable response to population change that protects human rights.

## Résumé

Confrontés à un déclin démographique important, de nombreux gouvernements ont recours à des politiques natalistes pour stimuler les taux de natalité, même si ces approches sont souvent inefficaces et risquent de porter atteinte aux droits reproductifs. La présente étude démontre qu’il existe une alternative politique plus efficace et plus immédiate: réduire la mortalité évitable et traitable. À partir des données des Nations unies, nous avons modélisé les projections démographiques jusqu’en 2050 dans 28 pays et territoires, en comparant un scénario de référence et deux critères: une augmentation immédiate du taux de fécondité au niveau de remplacement et la réduction des taux de mortalité nationaux pour les aligner sur ceux du Japon. Nos conclusions montrent que l’investissement dans la santé est un moyen plus efficace de réduire le déclin démographique que l’augmentation de la fécondité, en particulier dans les pays d’Europe de l’Est et du Sud-Est. Pour les pays de la région Europe de l’Organisation mondiale de la Santé les plus touchés par le déclin démographique, atteindre les niveaux de mortalité japonais permettrait de réduire de près de moitié la diminution de population. Cette variation serait de loin supérieure à la réduction du déclin attendue avec une approche fondée sur le taux de fécondité de remplacement. Nous considérons que la réduction de la mortalité doit constituer un pilier central de toute stratégie démographique. Cette approche offre des retombées démographiques plus rapides, s’aligne sur les objectifs en matière de droits humains et de vieillissement en bonne santé et offre un meilleur retour sur les investissements sociaux antérieurs dans l’éducation et la santé. Nous recommandons donc aux décideurs politiques de s’orienter vers le renforcement des systèmes de santé, la prévention des maladies et les interventions de santé publique. Dans le même temps, ils devraient intégrer ces mesures à des réformes institutionnelles plus larges afin d’apporter une réponse plus durable aux changements démographiques qui garantisse les droits humains.

## Resumen

Ante un marcado descenso de la población, muchos gobiernos han recurrido a políticas pronatalistas para aumentar las tasas de natalidad, aun cuando estos enfoques suelen ser ineficaces y pueden vulnerar los derechos reproductivos. Este estudio demuestra que existe una alternativa política más eficaz e inmediata: reducir la mortalidad prevenible y tratable. Utilizando datos de las Naciones Unidas, se realizó una modelización de las proyecciones demográficas hasta 2050 en 28 países y territorios, comparando un escenario basal con dos referencias: un aumento inmediato hasta la tasa de fecundidad de reemplazo y la reducción de las tasas nacionales de mortalidad hasta igualar las de Japón. Los resultados muestran que invertir en salud constituye una vía más eficaz para reducir el descenso de la población que aumentar la fecundidad, especialmente en los países de Europa oriental y sudoriental. En los países de la Región Europea de la Organización Mundial de la Salud que se ven más afectados por el descenso demográfico, alcanzar los niveles de mortalidad de Japón reduciría casi a la mitad la pérdida poblacional, superando ampliamente la disminución prevista con un enfoque basado en la fecundidad de reemplazo. Se considera que la reducción de la mortalidad debe ser un pilar central de la estrategia demográfica. Este enfoque ofrece beneficios demográficos más rápidos, se alinea con los objetivos de derechos humanos y de envejecimiento saludable, y proporciona un mayor rendimiento de las inversiones sociales previas en educación y salud. Por consiguiente, se recomienda que los responsables de formular políticas avancen hacia el fortalecimiento de los sistemas de salud, la prevención de enfermedades y las intervenciones de salud pública. Al mismo tiempo, deberían integrar estas medidas con reformas institucionales más amplias para lograr una respuesta más sostenible al cambio demográfico que proteja los derechos humanos.

## ملخص

في مواجهة الانخفاض الكبير في عدد السكان، لجأت العديد من الحكومات إلى سياسات مشجعة للإنجاب لرفع معدلات المواليد، على الرغم من أن هذه الأساليب غالبًا ما تكون غير فعالة ومن المحتمل ان تنتهك الحقوق الإنجابية. تشير هذه الدراسة لوجود بديل للسياسات أكثر فعالية وفورية: وهو خفض معدل الوفيات التي يمكن تجنبها وعلاجها. باستخدام البيانات المقدمة من الأمم المتحدة، قمنا بوضع نموذج للتوقعات السكانية حتى عام 2050 في 28 دولة ومنطقة، وقمنا بمقارنة سيناريو أساسي بمواجهة معيارين: زيادة فورية في معدل الخصوبة عند مستوى الإحلال، وخفض معدلات الوفيات الوطنية ليماثل نظيره في اليابان. تُظهر نتائجنا أن الاستثمار في الصحة هو وسيلة أكثر فعالية للحد من انخفاض عدد السكان، مقارنة برفع الخصوبة، لا سيما في دول شرق وجنوب شرق أوروبا. بالنسبة لدول منظمة الصحة العالمية، في المنطقة الأوروبية الأكثر تأثرًا بانخفاض عدد السكان، فإن تحقيق مستويات الوفيات في اليابان من شأنه أن يُقلل من الخسارة في السكان إلى النصف تقريبًا، وهو ما يفوق بكثير الانخفاض المتوقع في معدل الخصوبة عند مستوى الإحلال. نحن نعتبر أن خفض معدل الوفيات ينبغي أن يكون ركيزة أساسية في الاستراتيجية السكانية. يُحقق هذا الأسلوب فوائد سكانية أسرع، ويتماشى مع أهداف حقوق الإنسان والشيخوخة الصحية، ويُحقق عائدًا أقوى على الاستثمارات المجتمعية السابقة في التعليم والصحة. لذلك، نحن نوصي واضعي السياسات بالتحرك نحو تعزيز النظم الصحية، والوقاية من الأمراض، وتدخلات الصحة العامة. وفي الوقت نفسه، ينبغي عليهم دمج هذه الإجراءات مع إصلاحات مؤسسية أوسع نطاقًا من أجل استجابة أكثر استدامة للتغير السكاني، بحيث تحمي حقوق الإنسان.

## 摘要

面对人口骤减的局面，许多国家的政府转为推行鼓励生育的政策以期提高出生率，但是此类方法通常效果甚微且还有可能导致侵犯生殖权利。该项研究证明，可推行另外一种更加直接有效的政策：降低因可预防和可治疗疾病所致的死亡率。通过我们利用联合国所发布数据构建的截至 2050 年 28 个国家和领地的人口预测模型，我们基于以下两个基准情况对比了基线情景：更替率快速上升和全国死亡率下降（达到日本的死亡率水平）。研究结果表明，与提高生育率相比，投资于保障健康的方案能够更加有效地减缓人口下降趋势，特别是在东欧和东南欧国家。如果受人口下降影响最严重的世卫组织欧洲区域国家的死亡率水平能够与日本齐平，则人口减少规模将会削减大约一半，其效果远超更替率方法预期可实现的减缓人口下降。我们认为降低死亡率应当被视为人口战略的核心支柱。该方法能够更加快速地优化人口结构，符合人权和健康老龄化目标，并确保之前在教育和健康方面的社会投资获得更大的回报。因此，我们建议政策制定者着眼于优化卫生系统、强化疾病预防机制和加强公共卫生干预。同时，政策制定者应当将这些措施与更广层面的体制改革综合考虑，以针对人口变化采取有利于保护人权且更具持续性的应对方案。

## Резюме

Ввиду значительного сокращения населения правительства многих стран прибегают к политикам, поддерживающим рождаемость, несмотря на частую нерезультативность такого подхода и потенциальное ущемление репродуктивных прав. Согласно данному исследованию, существует более действенный способ с немедленным результатом: сокращение число смертей, которых можно избежать с помощью профилактики и лечения. Основываясь на данных ООН, авторы смоделировали прогноз роста населения до 2050 года для 28 стран и территорий, сравнивая базовые сценарии по двум параметрам: непосредственному росту рождаемости до уровня восполнения и сокращению национальных показателей смертности до уровня Японии. Полученные результаты свидетельствуют, что инвестиции в здравоохранение являются более эффективным способом сократить убыль населения, нежели попытки повысить рождаемость, что особенно актуально для государств Восточной и Юго-Восточной Европы. Для стран, относящихся к Европейскому региону Всемирной организации здравоохранения и наиболее сильно страдающих от сокращения численности населения, при достижении японских показателей смертности демографический спад сокращается практически вдвое, что намного превосходит результаты, которых можно было бы достичь с помощью выхода рождаемости на уровень восполнения. По мнению авторов, сокращение смертности должно быть основным элементом демографической стратегии. Такой подход обеспечивает более быстрый демографический эффект, не нарушает прав населения, соответствует целям здорового старения и означает более эффективное использование предыдущих общественных вложений в образование и охрану здоровья. В этой связи авторы рекомендуют политикам двигаться в направлении укрепления систем здравоохранения, профилактики заболеваний и действий, направленных на укрепление здоровья населения в целом. В то же время эти меры следует сочетать с более широкими институциональными реформами, чтобы реакция на изменения численности населения была устойчивой и соответствовала принципам защиты прав человека.

## Concern over population decline

Population decline is an important economic, social and political concern in many countries around the world,^1^ with some arguing that the future of health care is threatened by declining populations.^2^ Population decline is especially acute in the World Health Organization (WHO) European Region. All of the top 10 countries that are forecasted to see the greatest relative population decline between now and 2050 are in the WHO European Region, and primarily in eastern and south-eastern Europe (Republic of Moldova, Bosnia and Herzegovina, Lithuania, Bulgaria, Albania, Latvia, Serbia, Belarus, North Macedonia and Croatia, in order of relative magnitude of the decline).^3^ Each of these countries is projected to see its total population decline by between 18% and 23% by 2050.^3^

There is considerable debate over the perceived consequences of population decline. Some argue that population decline could lead to significant environmental benefits,^4^ improve access to housing and increase investment in human capital,^5^ which, in turn, could increase living standards.^6^ In contrast, the perhaps more usual view is that population decline will bring about a string of negative consequences for Europe’s existing economic frameworks, production systems and social welfare programmes.^6^^,^^7^ With a shrinking workforce, payroll tax income declines, thereby reducing funding for pensions and insurance schemes. At the same time, an ageing population increases demand for health care, elderly people care and retirement benefits. Additionally, population growth has long been postulated as a key factor in fostering technological advancement and business innovation as well as a perceived marker of geopolitical strength.^8^^,^^9^ It is usually argued that these changes will be more pronounced at the subnational level and in rural areas in particular.^10^

In this paper, we do not aim to make an in-depth exploration of the various consequences of population decline.^11^ Rather, we observe that it is a reality for many countries globally for the next few decades, especially in the WHO European Region, and that this reality will inevitably bring challenges as well as possibilities. In this paper, we focus more on the responses to these challenges.

## Holistic response or more babies?

As noted, population decline has wide-ranging consequences. Given the failure of pronatalist policies to increase fertility rates^12^ and potential ethical concerns about such policies, an interconnected and holistic set of policies will be required to improve health, increase productivity and reform strained institutions, such as pension, health and welfare systems. Indeed, many of the broader options within a comprehensive policy mix have been shown to be more effective at offsetting population decline and ageing. For example, increasing female participation in the labour force, increasing productivity through improvements in education and skills, and implementing migration policies that maximize the potential of migrants while reducing out-migration are all effective ways of reducing the demographic, economic and social effect of population ageing and population decline.^13^^–^^15^ Out-migration is especially important in eastern Europe.^16^ In addition, reform of strained pension, health-care and social security systems will be required, as well as general efforts to increase productivity and deliver education systems that give people of all ages the skills and tools they need to thrive in the current and future labour market.^17^^,^^18^

While such an integrated and holistic policy response is desirable, political reality means that more simplistic solutions are often followed. Furthermore, pension and health-system reforms are rarely popular with the voting public. Policy-makers in many areas have therefore sought a so-called demographic solution to a perceived demographic problem. Reluctant to increase immigration, many countries have opted to increase birth rates as a means of slowing population decline. Indeed, almost all countries with a total fertility rate lower than 1.5 children per woman have implemented policies aimed at increasing fertility.^12^ This approach is often (mis)perceived as a simple, politically convenient solution to population ageing and stagnation. While some family policy interventions have been implemented to support families and childbearing more broadly, many are explicitly designed to increase fertility towards a target birth rate. This target is usually the replacement rate of about 2.1 children per woman in advanced economies, but can be higher in places with higher mortality rates.^19^ Such pronatalist policies can be delivered through propaganda campaigns, direct cash transfers and other subsidies, or symbolic incentives such as giving special recognition to mothers with large families.

Some policies within a broad pronatalist set, such as expanded parental leave, flexible work arrangements and subsidized childcare, can have positive effects for family well-being. However, other policies are problematic and raise ethical concerns, particularly if they reduce access to sexual and reproductive health services, infringe on reproductive rights or impose or reinforce conservative gender norms.^12^^,^^20^ Furthermore, such policies, whether considered good or bad, are usually ineffective in their stated aim of changing the total number of children born; rather, they affect when children are born.^21^ These perceived failures can then fuel a negative cycle where the discourse around population decline becomes stronger, in turn potentially leading to ever-more radical pronatalist policies.

## More babies or fewer deaths?

Governments that fear population decline often compare the forecasted population with a scenario where the fertility rate jumps to replacement levels. Such a change, however unlikely, would without doubt slow the rate of population decline. As such, these hypothetical fertility scenarios are often used to justify pronatalist policies even if they infringe on reproductive rights and have other negative consequences.

Historically, improvements in health and decreased mortality have been drivers in population growth and, ultimately, population ageing. It has long been recognized that health promotion (including healthy ageing) is a vital component of ensuring that all people reach their full potential across the life course. This approach can in turn increase productivity and lessen dependence in older age, among other things.^22^^,^^23^ However, in the current discourse on population decline, while healthy longevity is widely discussed, the potential demographic impact of further reduction in mortality (that is, keeping more people alive) has barely featured. In many very low-fertility settings in eastern Europe, mortality (especially male mortality) is high in all age groups, with pronounced disparities at the subnational level. For example, in 2022, male life expectancy in Bulgaria (70.6 years), Latvia (69.4 years) and Romania (71.3 years) was 6–8 years lower than the European Union (EU) average of 77.9 years for men.^24^^,^^25^ Many of these deaths are avoidable. In the same year, 1.1 million people younger than 75 years in the EU died from preventable or treatable causes, with the highest rates observed in eastern Europe, particularly among men.^26^ Subnational mortality inequalities are also evident in Latvia and Hungary; for instance, life expectancy in rural or resource-constrained regions was about 2 years lower than in urban areas.^26^

We therefore propose an alternative (albeit ambitious) benchmarking scenario: instead of having more babies, what would be the effect of keeping more people alive longer? More precisely, what would be the effect on overall population change of focusing on improving health and reducing mortality rather than on increasing fertility?

## Estimates based on fewer deaths

To answer these questions, we produced a series of population forecasts based on the 2022 United Nations (UN) population projections.^27^ We made calculations for all countries with a population greater than 1 million to determine the extent to which mortality reduction is a more successful way of slowing population decline than increasing fertility. To make our alternative projections, we applied the cohort-component projection method, a widely used demographic technique that incorporates age-specific fertility and mortality rates. We used two simple benchmark scenarios to compare with the UN medium variant (baseline). The first scenario assumed an instant shift to Japan’s age-specific mortality rates (representing a so-called healthy future). In this projection, we assumed the mortality rate immediately dropped to match and follow the rate in Japan, currently the country with the highest life expectancy at birth (more than 84 years). In the second scenario, we used a UN projection based on the original mortality assumption but assumed fertility immediately jumped to replacement rate of fertility, a figure that many policy-makers desire. Both projections assumed zero migration.^27^ We compared the impact on population decline of such a large shift in mortality to the impact of a shift in fertility.

The projection assumptions are shown in Table 1. Neither model is a forecast as such; rather they are two theoretical yardsticks for policy comparison. Furthermore, we used Japan only as a mortality benchmark, not a policy template. Indeed, it is important to acknowledge Japan’s unique demographic situation, characterized by its historically low fertility and rapid ageing, which presents its own set of challenges. 

**Table 1:** Assumptions and inputs for three population forecast scenarios

Scenario	Fertility	Mortality	Migration	Rationale
Baseline: UN medium	Country-specific UN medium	Country-specific UN medium	0	Diagnostic baseline consistent with the 2022 world population prospects,^27^ projected natural increase
Healthy: Japan mortality	Country-specific UN medium	Set to Japan age-specific rates from 2022 onwards	0	Upper benchmark for mortality improvement to estimate the greatest impact of reduced mortality on population decline
Instant replacement fertility	Country-specific UN estimates of precise replacement rate (e.g. 2025 total fertility rate range was 2.05–2.60)	Country-specific UN medium	0	Replacement fertility rate to estimate impact of meeting this target fertility level on population decline

UN: United Nations.

## Impact of mortality reduction

Our full results are given in Table 2 and in the online repository.^28^ They show that at least 28 countries and territories with a population of more than 1 million would experience a smaller reduction in population if they increased lifespan rather than fertility rates.

**Table 2:** Fertility rate, life expectancy, and projected population of countries and territories to 2050, by scenario

Country or territory^a^	Total fertility rate, children per woman^b^	Life expectancy, years^b^			Population, in millions^c^			
		Males	Females		UN estimates for 2021	(0) UN 2050	(1) 2050, Japan mortality	(2) 2050, instant replacement rate fertility
Albania	1.39	74.1	79.2		2.86	2.74	2.89	3.10
Argentina	1.89	72.2	78.6		45.16	51.49	54.46	54.21
Armenia	1.58	66.6	77.4		2.80	2.75	3.05	3.02
Australia	1.60	83.2	85.8		25.80	27.12	27.28	29.18
Austria	1.47	79.0	84.1		8.91	8.23	8.43	8.95
Azerbaijan	1.66	65.6	73.3		10.30	10.88	12.19	11.96
Bahrain	1.81	77.8	80.0		1.46	1.70	1.74	1.79
Bangladesh	1.98	70.6	74.3		168.41	213.47	228.14	225.78
Belarus	1.48	67.3	77.7		9.61	8.36	9.54	9.07
Belgium	1.58	79.4	84.3		11.58	11.24	11.48	12.07
Bosnia and Herzegovina	1.35	73.1	77.5		3.30	2.77	3.10	3.10
Brazil	1.64	69.6	76.0		213.83	230.86	248.38	251.91
Bulgaria	1.59	68.4	75.5		6.94	5.45	6.29	5.88
Canada	1.46	80.6	84.7		38.02	36.87	37.31	40.42
Chile	1.54	76.5	81.4		19.39	20.70	21.17	22.68
China	1.16	75.5	81.2		1425.86	1327.43	1412.81	1511.09
China, Hong Kong SAR	0.75	82.7	88.3		7.50	6.31	6.26	7.52
China, Taiwan	1.11	77.8	84.4		23.84	21.69	22.43	24.58
Colombia	1.72	69.4	76.4		51.24	58.49	61.62	63.46
Costa Rica	1.53	74.4	79.8		5.14	5.62	5.80	6.21
Croatia	1.45	74.2	81.1		4.08	3.42	3.71	3.76
Cuba	1.44	71.2	76.4		11.29	10.27	11.11	11.28
Cyprus	1.32	79.2	83.2		1.24	1.22	1.25	1.36
Czechia	1.70	74.7	80.9		10.52	9.51	10.19	10.06
Democratic People’s Republic of Korea	1.81	70.8	75.7		25.92	25.92	29.29	27.75
Denmark	1.72	79.5	83.3		5.84	5.71	5.88	6.05
Ecuador	2.03	70.3	77.5		17.69	22.46	23.43	23.39
El Salvador	1.80	66.1	75.1		6.30	7.42	8.15	8.04
Estonia	1.68	72.8	81.2		1.33	1.21	1.29	1.29
Finland	1.39	79.3	84.7		5.53	4.98	5.12	5.50
France	1.79	79.4	85.5		64.50	63.69	64.87	66.72
Georgia	2.08	66.8	76.7		3.76	3.75	4.25	3.85
Germany	1.53	78.1	83.2		83.39	73.55	75.88	79.59
Greece	1.37	77.5	82.9		10.48	9.00	9.37	9.95
Hungary	1.58	71.1	77.9		9.73	8.35	9.29	9.04
India	2.03	65.8	68.9		1402.81	1685.75	1848.91	1768.45
Iran (Islamic Republic of)	1.69	71.2	76.8		87.59	100.35	105.89	108.03
Ireland	1.77	80.2	83.8		4.97	5.36	5.44	5.66
Italy	1.28	80.5	85.1		59.36	50.40	50.97	55.85
Jamaica	1.35	68.5	72.5		2.83	2.87	3.26	3.30
Japan	1.30	81.8	87.7		124.95	100.66	100.41	111.09
Kosovo (in accordance with Security Council resolution 1244 (1999))	1.52	74.2	79.6		1.67	1.85	1.95	2.05
Kuwait	2.11	77.2	81.5		4.25	4.75	4.90	4.84
Latvia	1.58	69.2	77.8		1.89	1.53	1.76	1.66
Lebanon	2.09	72.8	77.3		5.63	6.84	7.31	7.04
Lithuania	1.62	68.8	78.8		2.80	2.36	2.66	2.52
Malaysia	1.80	72.7	77.4		33.40	39.23	41.85	42.01
Mauritius	1.41	70.4	76.8		1.30	1.23	1.37	1.39
Mexico	1.82	66.1	74.9		126.39	147.43	159.34	158.20
Nepal	2.03	66.6	70.4		29.70	39.27	42.31	41.37
Netherlands (Kingdom of the)	1.64	80.0	83.4		17.47	16.83	17.27	18.03
New Zealand	1.77	80.6	84.3		5.10	5.48	5.52	5.82
North Macedonia	1.36	71.7	76.2		2.11	1.95	2.12	2.18
Norway	1.59	81.6	84.9		5.39	5.36	5.43	5.83
Poland	1.46	72.6	80.4		38.38	33.88	36.80	37.09
Portugal	1.36	77.8	84.1		10.30	8.94	9.20	9.90
Puerto Rico	1.29	75.9	84.5		3.26	2.75	2.92	3.11
Qatar	1.80	78.3	80.49		2.69	2.99	3.03	3.11
Republic of Korea	0.88	80.4	86.8		51.83	45.00	45.82	52.84
Republic of Moldova	1.81	64.4	73.5		3.07	2.85	3.39	3.02
Romania	1.75	70.6	77.9		19.39	17.36	19.24	18.40
Russian Federation	1.49	64.2	74.8		145.47	129.66	148.55	140.00
Serbia	1.53	71.2	77.2		7.33	6.15	6.86	6.71
Singapore	1.02	80.6	84.9		5.93	5.43	5.47	6.22
Slovakia	1.57	71.5	78.4		5.46	4.97	5.40	5.37
Slovenia	1.63	77.6	83.8		2.12	1.94	2.00	2.06
Spain	1.28	80.2	85.8		47.40	42.96	43.19	47.81
Sri Lanka	1.99	73.1	79.5		21.75	24.62	26.55	25.58
Sweden	1.67	81.1	84.9		10.42	10.44	10.48	11.12
Switzerland	1.49	82.0	85.9		8.67	8.32	8.35	9.01
Thailand	1.33	74.5	83.0		71.56	67.44	70.92	76.15
Trinidad and Tobago	1.63	69.7	76.4		1.52	1.51	1.68	1.65
Tunisia	2.09	70.7	77.1		12.22	14.43	15.30	14.87
Türkiye	1.89	73.0	79.1		84.46	100.28	104.71	105.35
Ukraine	1.25	66.5	76.7		43.73	35.73	41.13	40.21
United Arab Emirates	1.46	77.2	80.9		9.33	10.24	10.38	11.01
United Kingdom	1.56	78.7	82.8		67.17	65.79	67.42	71.07
United States of America	1.66	74.3	80.2		336.50	338.88	356.10	362.82
Uruguay	1.49	71.7	79.3		3.43	3.39	3.63	3.78
Viet Nam	1.94	69.1	78.2		97.09	109.73	119.62	114.60

SAR: Special Administrative Region; UN: United Nations.

^a^ We included countries and territories with a population more than 1 million and a total fertility rate in 2022 of ≤ 2.1.

^b^ Data source: UN, 2022 ^27^

^c^ UN estimates for 2021;^27^ scenario 0 gives projected estimates with zero migration based on UN medium variant; scenario 1 gives projected estimates with zero migration assuming Japanese mortality rates; and scenario 2 shows projected estimates with zero migration assuming immediate jump to replacement rate fertility.

**Fig. 1:**
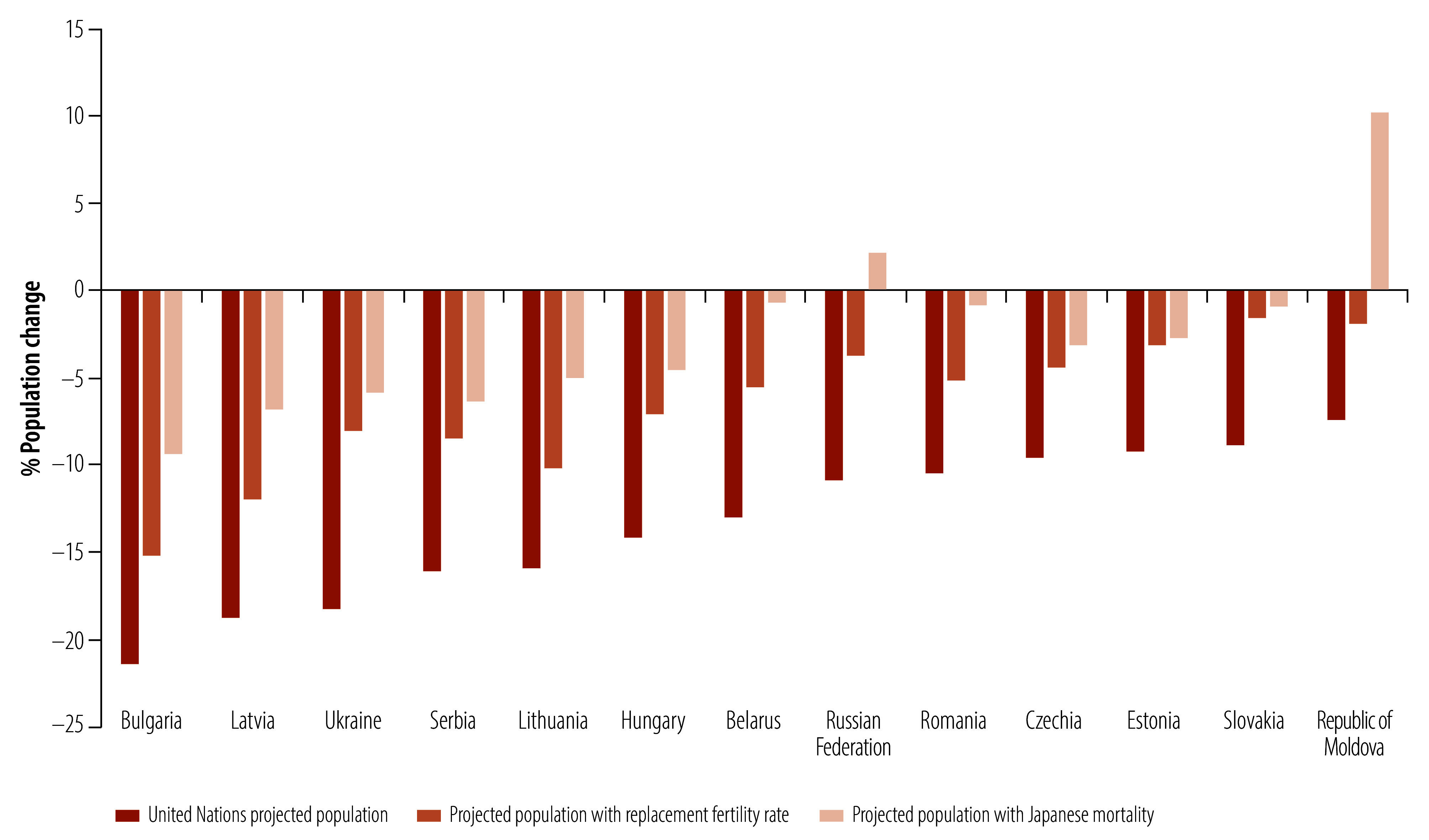
Estimated percentage change in total population between 2021 and 2050 with no intervention, immediate increase to replacement fertility rate and reduction in mortality, WHO European Region

Fig. 1 shows the 13 countries in the WHO European Region that are projected to see a population decline of 5% or more by 2050. The figure shows that reducing mortality would have a stronger effect on total population than an immediate increase to the replacement rate fertility. As expected, Fig. 1 and Table 2 show that the impact on population size of reducing mortality is greatest in countries with low fertility and relatively high mortality levels. It is immediately apparent from the online repository^28^ and Fig. 1 that these countries are predominantly in eastern and southern Europe, and were either part of the former Soviet Union or satellite states, or were part of former Yugoslavia. The legacy of higher mortality in these states means that gains from improved health in these countries today and in succeeding decades could have a substantial demographic impact.^21^

Together, these countries are projected to see their population decline from 267.3 million in 2022 to 233.5 million in 2050. In response, many of these countries have introduced policies with strong pronatalist elements and, in some cases, have sought to stigmatize childlessness and reduce access to sexual and reproductive health services.^29^ As Fig. 1 shows, an immediate (and highly unlikely) jump to the replacement fertility rate would, if held constant to 2050, result in a population of 253.2 million. However, if these countries were to see improved health and transition to very low mortality rates, the total population by 2050 would be 265.6 million. This fall represents a decline of only 1.7 million people under the mortality-improvement scenario, compared with declines of 33.8 million with no intervention and 14.1 million under the replacement fertility rate scenario. In fact, in two cases, Republic of Moldova and Russian Federation, even a sharp, highly unlikely jump to the replacement rate fertility rate would not prevent continued population decline, while a substantial decrease in mortality would result in population increases.

While our findings provide a clear empirical demonstration of the potential demographic impact of decreases in mortality, it is important to acknowledge the limitations of our exercise. The scenarios presented here are intentionally designed to benchmark the relative effects of two policy pathways and offer a useful comparative perspective. As such, they do not account for the considerable uncertainty inherent in all population forecasts, particularly regarding future trends in mortality, fertility and migration. We also did not model the effects of combined interventions, for example, investments in health alongside changes in migration or family policy, which are directions for future research. Furthermore, while reduction in mortality is strongly associated with improved health, the relationship is complicated and future trends in morbidity and disability require careful monitoring.

Despite these limitations, these results clearly demonstrate that investment in improving health and reducing mortality can lead to stronger demographic outcomes than pronatalist measures. 

## Alternative to slow the decline

Population decline, particularly when coupled with population ageing, presents profound challenges to the sustainability of our health, welfare and economic systems.^2^^,^^7^ While a holistic response involving substantial institutional reform is essential,^13^^,^^15^ many governments have disproportionately relied on pronatalist policies as a seemingly straightforward solution.^12^^,^^19^ Not only do these measures often fail to achieve sustained fertility increases, they also have important social risks, from reinforcing restrictive gender norms to fuelling ethnonationalist viewpoints.^11^^,^^20^^,^^30^^–^^32^

Our analysis shows that a more effective and ethically sound alternative is possible. In the European context studied here, we demonstrate empirically that reducing mortality is a more powerful way to slow population decline than raising fertility. Even partial progress, far short of matching global leaders in mortality such as Japan, would produce significant demographic benefits, probably more than the marginal gains from fertility subsidies. Increasing healthy lifespan is not a theoretical prospect; several countries have achieved rapid gains in life expectancy through improved health-care access, public health initiatives and medical innovation.^33^ In contrast, the evidence that pronatalist policies can reliably increase fertility is weak,^20^^,^^21^ a reality acknowledged by countries such as Republic of Moldova, which has shifted its focus towards gender-responsive and work-life balance policies.^34^

A common objection to reducing mortality, especially at older ages, is that it will worsen population ageing. While it is true that population ageing brings important challenges regarding, for example, health and social welfare systems, such a perspective is both ethically problematic and demographically narrow.^35^ The objection implicitly devalues the lives of older persons. Furthermore, conventional measures of ageing often overstate its economic burden.^36^^,^^37^ The economic and social contributions of older persons to society are well documented. Across the Organisation for Economic Co-operation and Development, for example, the labour force participation rate for the age group 65–69 years is 30% and has risen sharply in recent years.^38^ Older persons contribution to the economy overall, as well as the care economy in particular, has led to the coining of the term second and third demographic dividends.^39^

Reducing preventable mortality alone will not solve all challenges, but when coupled with policies that promote healthy ageing, such as the Madrid International Action Plan for Ageing and the WHO Decade of Healthy Ageing, this approach can help societies realize the benefits of longer, healthier lives. By reducing preventable mortality at working and early older ages, fostering age-friendly environments and combatting ageism, populations do not just live longer and healthier lives, but are also more capable of continuing to contribute actively to society and the economy.^40^^,^^41^

From a policy perspective, investing in mortality reduction offers many different and immediate advantages. The impact on population size is direct and swift, unlike the decades-long lag in fertility policies. While immigration also has an immediate effect, political resistance often limits its scope.^42^^–^^44^ Importantly, saving lives ensures that the substantial investments in an individual’s education and health are fully realized, contrasting with the delayed and uncertain returns of fertility-focused strategies.

Therefore, we argue that reducing treatable and preventable mortality must be a core component of any demographic strategy tackling population decline. This approach requires targeted investment in strengthening primary and community-based care; preventing and controlling noncommunicable diseases; improving screening and treatment for cardiovascular disease and cancer; enhancing mental health services and suicide prevention; and ensuring financial protection to reduce unmet health-care needs.^41^^,^^45^^,^^46^ Indeed, in eastern Europe where the highest impact of lowering mortality is seen, further improvements in preventable deaths from alcohol abuse, smoking and poor diet, particularly among men, should be prioritized.^47^^,^^48^

This focus on health-centred policies does not mean ignoring fertility entirely; rather, fertility-related policies should be based on the agenda of the International Conference on Population and Development, that is, ensuring sexual and reproductive rights, enabling free and informed choices, and supporting individuals’ own reproductive goals through gender-responsive and family-friendly policies.^49^^,^^50^

In conclusion, our paper provides an important empirical tool to challenge the anxiety-driven views that justify target-based pronatalism. We have shown that reducing mortality has a greater impact on population size than increasing fertility, thereby negating a main justification for potentially harmful policies. By prioritizing health and longevity, we can not only slow or reverse patterns of population decline but also build a foundation for greater well-being for all. While our study focuses on countries in the WHO European Region, the imperative to invest in human life and health is a universal lesson for all nations facing a future of demographic change.
